# Achieving HIV-1 Control through RNA-Directed Gene Regulation

**DOI:** 10.3390/genes7120119

**Published:** 2016-12-07

**Authors:** Vera Klemm, Jye Mitchell, Christina Cortez-Jugo, Francesca Cavalieri, Geoff Symonds, Frank Caruso, Anthony Dominic Kelleher, Chantelle Ahlenstiel

**Affiliations:** 1 Kirby Institute, The University of New South Wales, Sydney, NSW 2052, Australia; vklemm@kirby.unsw.edu.au (V.K.); jmitchell@kirby.unsw.edu.au (J.M.); akelleher@kirby.unsw.edu.au (A.D.K.); 2 ARC Centre of Excellence in Convergent Bio-Nano Science and Technology, The University of Melbourne, Parkville, Victoria 3010, Australia; christina.cortez@unimelb.edu.au (C.C.-J.); francesca.cavalieri@unimelb.edu.au (F.C.); fcaruso@unimelb.edu.au (F.C.); 3 Department of Chemical and Biomolecular Engineering, The University of Melbourne, Parkville, Victoria 3010, Australia; 4 Calimmune Inc., Darlinghurst, NSW 2011, Australia; geoff.symonds@calimmuneinc.com

**Keywords:** HIV-1, RNAi, transcriptional gene silencing, gene therapy, clinical trials

## Abstract

HIV-1 infection has been transformed by combined anti-retroviral therapy (ART), changing a universally fatal infection into a controllable infection. However, major obstacles for an HIV-1 cure exist. The HIV latent reservoir, which exists in resting CD4+ T cells, is not impacted by ART, and can reactivate when ART is interrupted or ceased. Additionally, multi-drug resistance can arise. One alternate approach to conventional HIV-1 drug treatment that is being explored involves gene therapies utilizing RNA-directed gene regulation. Commonly known as RNA interference (RNAi), short interfering RNA (siRNA) induce gene silencing in conserved biological pathways, which require a high degree of sequence specificity. This review will provide an overview of the silencing pathways, the current RNAi technologies being developed for HIV-1 gene therapy, current clinical trials, and the challenges faced in progressing these treatments into clinical trials.

## 1. Introduction

HIV-1 currently infects more than 37 million people worldwide. The care of patients with HIV-1 infection and access to treatment has been transformed by combined antiretroviral therapy (ART), changing a universally fatal infection into a chronic controllable infection. Adherent patients, treated according to current guidelines, have predicted actuarial survival of 30–40 years from commencing therapy [1]. However, drug treatment is for life and there is residual mortality and morbidity.

The current World Health Organisation (WHO) guidelines, updated in 2015 [2] largely based on the findings from randomised clinical trials, START (Strategic Timing of Antiviral Treatment) [3] and TEMPRANO (TEMPRANO ANRS 12136) [4], recommend that ART is commenced in all individuals with HIV-1 infection, regardless of disease stage [2]. These recommendations have increased the number of people eligible for ART from 15.8 million to all 37 million currently living with HIV. This has created an enormous logistic and economic challenge, as drug treatment is currently life-long. Alternate treatments to ART currently being explored involve RNA-directed gene regulation in the form of gene therapy approaches.

The field of RNA interference (RNAi) has expanded exponentially since its discovery in 1998 by Fire and Mello [5]. This study identified the post-transcriptional gene silencing pathway (PTGS), which predominately occurs in the cytoplasm, and results in mRNA degradation via target sequence complementarity. Another RNA pathway, termed transcriptional gene silencing (TGS), was identified in 2004 and shown to function in the nucleus via target sequence complementarity to the gene promoter region, which induced epigenetic silencing of the gene [6]. Both of these naturally-occurring RNA pathways, PTGS and TGS, have been utilised in the development of potential therapeutics against a range of diseases, including HIV-1.

This review highlights current advances in RNA therapeutics, particularly short interfering RNA (siRNA) and short hairpin RNA (shRNA), and gene-editing systems, including zinc finger nucleases (ZFN) and CRISPR/Cas9 or dCas9, which are being used or have potential for use in gene therapy approaches to treat HIV-1 infection. We will also discuss developments in delivery, which represents a major challenge for progressing these RNA treatments forward into clinical trials.

## 2. The Latent Reservoir

Sustained disruptions in ART during chronic HIV infection are associated with rapid return of plasma viraemia to pre-therapy levels in the overwhelming majority of patients, as well as increased morbidity and mortality [7,8]. Viral recrudescence arises from integrated provirus and, while ART reduces the extent of proviral, its effects on this form of the virus are relatively limited [9,10,11,12,13]. The integrated provirus forms a viral reservoir, known as the latent reservoir, which resides predominantly in long-lived resting T cells, tissue-based macrophages, myeloid cells in the CNS, and other sanctuary sites [14,15]. It is thought the majority of the virus reservoir is in a latent form [16], mediated by a series of epigenetic mechanisms [17]. Virus in the latent reservoir has the potential to reactivate from latency upon activation of these cells by antigen or changes in the local inflammatory milieu [18,19]. Thus, this source of virus represents the major barrier to curing HIV. A multitude of approaches have been explored to address the challenge of eradicating the latent reservoir, including early commencement of ART [10], intensifying ART regiments [20,21,22,23,24,25] and attempts to purge the reservoir using various reactivation strategies [26,27,28,29,30], however, none have substantially reduced the extent of rebound virus in the majority of patients following ART interruption [31].

## 3. HIV Cure: Sterilising or Functional?

The aspirational pinnacle of HIV treatment is achieving infection control without the need for ART. This requires reservoir control. Most efforts impacting the reservoir have been aimed at eradicating integrated provirus, thereby achieving a sterilizing cure, defined as purging the reservoir of all latent virus and an undetectable plasma viral load, without requiring ART. This approach has been the most extensively studied, and is termed the “Shock and Kill” approach. The underlying hypothesis to this approach is that after HIV-1 infected cells are reactivated by various stimuli they apoptose or are killed by cytotoxic T cells following expression of foreign viral proteins on the cell surface. In theory this approach is warranted. However, numerous studies involving multiple stimuli, such as IL-2 [17], IL-7 [29,32], OKT3-induced pan T cell activation [33] and activation of protein kinase C pathway, with drugs including prostatin, bryostatin, or disulfram [34] have reported very limited success in reducing the latent reservoir. Studies have also reported on reactivation of virus using a suite of histone deacetylase (HDAC) inhibitors to change the latent reservoir epigenetic profile. In vitro studies in cell lines treated with trichostatin A (TSA) and vironostat (SAHA), have shown successful reactivation of the latent virus [35,36,37]. However, ex vivo studies of valproic acid, vironostat, panobinostat, and rombidepsin have shown limited virus reactivation, with non-specific drug effects observed in host genes [27,38,39,40,41]. Similarly, ex vivo studies have shown potent reactivation by protein kinase C agonists [42,43,44], although this approach is limited, as not all cells within the reservoir become reactivated [45] and those that are reactivated do not illicit a robust cytotoxic T lymphocyte immune response and survive [46], presenting a source of infectious virus.

A clinical trial (NCT01365065) reported in 2014 by Elliott et al. showed a short 14 day course of vironostat, in 20 HIV-infected patients receiving suppressive ART, was sufficient to significantly increase levels of cell associated unspliced HIV RNA in 90% patients, with a median fold change of 7.4 compared to baseline [47]. The study did not report any significant changes in immune activation or reduction of integrated virus, indicating supplementary interventions will be required to achieve this [47]. Another clinical trial reported in 2014 by Rasmussen et al. [48] investigated the effect of panobinostat in 15 HIV-infected adults receiving ART (NCT01680094) and demonstrated similar results to vironostat treatment, with significant 3.5-fold increases in cell-associated unspliced HIV RNA compared to baseline, but no changes to the latent reservoir, again suggesting additional interventions are required for this effect. The most recent clinical trial investigating romidepsin (NTC02092116) treatment in HIV-1 infected adults has also reported HIV-1 transcription is induced to low, but readily quantifiable, levels that were statistically significant increases compared to baseline. These changes in cell associated viral RNA and plasma viral load correlated with the timing of increases in the degree of global lymphocyte histone H3 acetylation [49]. The treatment was safe, with adverse events (all grade 1–2) being consistent with side effects of drug treatment and importantly, did not change the proportion of HIV-specific T cells or inhibit T cell cytokine production [49]. These are critical features for potential clinical trials of combining HDAC inhibitors with interventions such as a therapeutic HIV-1 vaccine.

In view of the limited progress in developing HDAC inhibitors, a potentially more specific and targeted approach to controlling the latent reservoir is represented by a functional cure strategy for HIV, defined as maintaining control of the reservoir without its eradication, and an undetectable plasma viral load, without requiring ART. Studies exploring this approach involve the use of gene therapy to introduce therapeutics that protect against new HIV infection or reactivation of existing infection, most of which include, but are not restricted to, RNA therapeutics that inactivate viral and/or down-regulate host targets and will be discussed in detail below.

## 4. RNA Silencing Pathways

**Figure 1:**
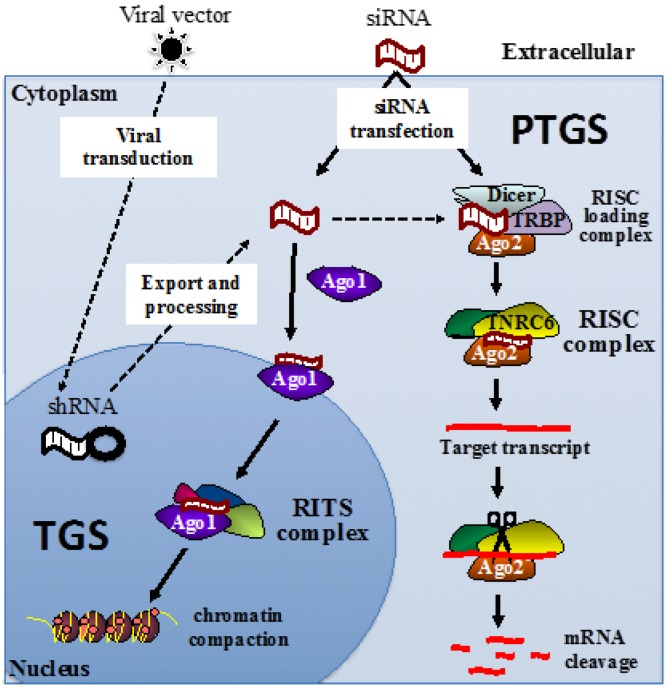
Mechanisms of siRNA-induced gene silencing. siRNA duplexes induce transcriptional gene silencing (TGS) in the nucleus via the RITS complex initiating epigenetic modifications, and post-transcriptional gene silencing (PTGS) via RISC machinery initiating specific mRNA cleavage in the cytoplasm. (Ago1: Argonaute 1, Ago2: Argonaute 2, RISC: RNA-induced silencing complex, RITS: RNA-induced transcriptional silencing complex, shRNA: short hairpin RNA, TRBP: transactivating response (TAR) RNA-binding protein, and TNRC6: trinucleotide repeat containing six protein).

While PTGS has become a mainstream tool for siRNA knockdown of mRNA expression, the TGS pathway is slowly being recognized as an important biological process for inducing and controlling epigenetic profiles of targeted genes. The two pathways are quite distinct, as shown in Figure 1. PTGS primarily occurs in the cytoplasm, although some exceptions have been reported [50,51]. TGS occurs only in the nucleus at the site of the gene promoter [6,52,53,54]. Both pathways require an Argonaute (Ago) protein to facilitate RNA binding. In the case of PTGS, the Ago2 protein binds the siRNA guide strand sequence [55,56,57], which is loaded by the RNA-inducing silencing complex (RISC)-loading complex comprising of the Dicer RNase III endonuclease protein; TRBP, transactivation response (TAR) RNA-binding protein; and the double stranded siRNA. This complex is also essential for microRNA (miRNA) biogenesis and processing into a RISC-mediated gene silencing pathway that functions via translational or transcriptional repression of mRNA [58]. Once loaded by the RISC-loading complex, the Ago2 and siRNA form the RISC [59], which includes the trinucleotide repeat containing six protein, known as TNRC6 [60]. The catalytic activity in Ago2 then cleaves the complementary mRNA sequence target, resulting in silencing of the mRNA [55].

In the TGS pathway, Ago1 is essential for siRNA binding in the nucleus [61], which then forms the RNA-induced transcriptional silencing (RITS) complex. The components of RITS in mammalian cells are still unclear, however in fission yeast *Schizosaccharomyces pombe*, the RITS complex is comprised of Ago1, Chp1 (a heterochromatin-associated chromodomain protein) [62,63], Tas3 (a novel protein) [64,65], and siRNA [61]. Unlike Ago1 in fission yeast, human Ago1 does not have the catalytic ability of Ago2, but does facilitate recruitment of other proteins to induce epigenetic modifications initially at the complementary sequence in the promoter region. There are many epigenetic modifications that can occur, however the major modifications include DNA methylation or histone modifications. DNA methylation frequently occurs at CpG sites, where DNA cytosine methyltransferases, (e.g., DNMT1, 3a and 3b) can add methyl groups to the cytosine, which changes the architecture of the DNA major groove to prevent the binding of DNA binding proteins, such as transcription factors. Promoters are frequently rich in CpG islands and, thus, promoter methylation is commonly used to repress initiation of transcription. Another set of enzymes, such as histone deacetylases and histone methyltransferases, can be recruited by the downstream effects of RITS, to modify specific amino acid residues within histones. In general, increased methylation and decreased acetylation of Lysines 9 and 127 of histone 3, help generate compaction of chromatin, generating heterochromatin [66].

## 5. RNA Therapeutics Targeting HIV by PTGS

Although PTGS is a mainstream tool for gene knockdown, a limitation of the pathway, in the context of control of HIV replication, is the opportunity for virus escape mutants to arise in target mRNA through mutations incorporated during the transcription and reverse transcription processes [67,68,69]. Due to its high level of sequence specificity, single or dual nucleotide substitutions can disable this process by changing the target sequence. One strategy used to compensate for this inherent feature of PTGS is to use multiple combinations of anti-HIV shRNAs alone or further combined with other anti-HIV therapeutics, mimicking the approach of combined ART to prevent HIV drug resistance [70,71,72].

The first HIV-1 gene therapy treatment to progress to a phase 2 clinical trial was a combination approach employing a *tat-vpr*-specific anti-HIV ribozyme, called OZ1, which was delivered in transduced autologous CD34+ HSC [73]. This study demonstrated the RNA therapeutic was safe and efficacious, with CD4+ lymphocyte counts being higher in the OZ1 treated group compared to the placebo group throughout the 100 week trial, despite there being no statistically significant differences in viral load reported at the primary end point of weeks 47–48 [73].

The next pilot clinical trial of an HIV-1 combination gene therapy occurred in HIV-1 infected patients with AIDS-associated lymphoma and utilized a triple therapy comprising *tat-rev* shRNA, a TAR decoy, and a *CCR5* ribozyme. The RNA therapeutic was delivered using lentiviral transduction of CD34+ HSC and was shown to be safe with no adverse events reported. However, there was also no therapeutic effect observed, despite detection of the delivered RNA and ribozyme in peripheral blood mononuclear cells (PBMC) and bone marrow for up to eight months in all four patients receiving treatment and greater than three years in one of those patient [72].

This approach was further optimised to comprise three shRNAs, targeting HIV integrase, protease and *tat-rev* genes, which were delivered and expressed in a single lentivirus vector called R3A [74]. This study was instrumental in demonstrating that equivalent expression of multiple shRNAs was possible using a single lentiviral vector, but was highly dependent on each shRNA′s expression being controlled by a unique promoter [74]. Preclinical data of this construct in an in vivo study using the Balb/c Rag2(−/−) IL-2Rγc(−/−) (BRG) humanized mouse model showed the RNA therapeutic was safe [75]. Additional optimization of this approach has resulted in a lentiviral construct expressing a combination of four anti-HIV agents, including three anti-HIV RNAs expressed from an intronic MCM7 (minichromosome maintenance complex component-7) platform and a CCR5 shRNA [76,77].

One of the most exciting combination HIV gene therapies currently in phase I/II trials (NCT01734850), developed by Calimmune Inc. (Pasadena, CA, USA), is a dual anti-HIV-1 lentiviral vector, termed Cal-1, which comprises a CCR5 shRNA and the C46 fusion inhibitor [70,71,78,79]. Cal-1 is delivered via lentiviral transduction of CD34+ HSC and CD4+ T cells and protects against both CCR5-tropic and CXCR4-tropic HIV-1 strains, due to the shRNA and C46 fusion inhibitor, respectively. Preclinical data shows the Cal-1 treatment is safe and has no adverse effects on HSC differentiation [79]. Additional studies in a humanized bone marrow, liver, thymus (BLT) mouse model demonstrated human CD34+ HSC transduced with Cal-1 and transplanted into animals displayed similar engraftment and multi-lineage hematopoietic differentiation as untransduced animals. Importantly, only the Cal-1 treated animals displayed significant protection of CD4+ T cells after challenge with R5-tropic HIV-1, as shown by reduced viral load in peripheral blood and lymphoid tissues. Ex vivo experiments in splenocytes isolated from the Cal-1 treated animals also showed resistance to both R5- and X4-trpic HIV-1 strains [71]. Further, the safety and efficacy of Cal-1 was recently evaluated in pigtailed macaques (*Macaca nemestrina*) infected with simian/human immunodeficiency disease (SHIV), a nonhuman primate model of AIDS. Animals transplanted with autologous CD34+ HSC transduced with Cal-1 demonstrated robust gene marking in myeloid and lymphoid lineages and no measureable adverse effects. This result provides strong preclinical evidence for safety and efficacy of Cal-1 treatment, which generates multi-lineage engraftment following myeloablative conditioning [78]. Taken together, the in vitro and in vivo studies of Cal-1 provide strong support for its use in cell modified gene therapy applications for inhibiting HIV-1 infection.

## 6. RNA Therapeutics Targeting HIV by TGS

The TGS pathway provides significant advantages over a PTGS approach, specifically with respect to HIV treatment. Since TGS acts directly at the gene promoter to induce epigenetic silencing, there is a more limited opportunity for virus escape to occur, which we have concluded through cloning and sequencing many provirus, but not observing virus escape at MOIs of 0.1 to 100 [80]. Another key advantage is the epigenetic profile of a silenced promoter is heritable and can be passed on and maintained in the next cell generation. 

**Figure 2:**
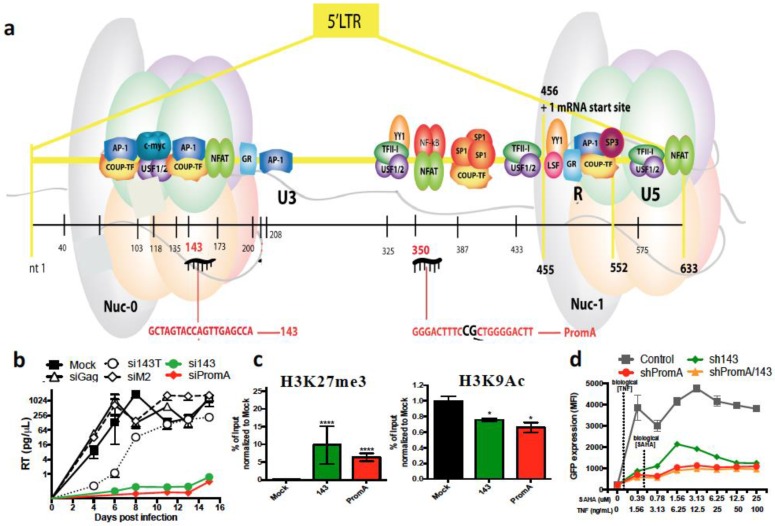
siPromA and 143 potently suppress virus transcription. (**a**) Viral sequences targeted within HIV-1 5 transcrsiPromA and si143; (**b**) SiPromA- and 143-transfected cultures show suppression of virus transcription 15 days post-infection; (**c**) heterochromatin marks observed in siPromA- and si143-transfected cells suppressing HIV-1_SF162_ infection by ChIP analysis showed enrichment of H3K27me3 and reduction of H3K9Ac; and (**d**) J-Lat 9.2 cells transduced with dual shPromA and 143 are less susceptible to combined vironostat/TNF reactivation as shown by limited GFP expression upon activation.

Suzuki and colleagues identified the first HIV-1 promoter-targeted TGS-inducing siRNA, termed PromA, in our laboratory in 2005. The HIV-1 promoter region targeted by the siRNA sequence was the tandem repeat of NF-κB binding motifs in the 5′ LTR (Figure 2a), which has also been of interest in other HIV studies due to its potent transcriptional activation of the virus [81,82,83]. We have demonstrated profound silencing of HIV-1 replication up to 1000-fold from a single treatment of siRNA or shRNA delivered by lentivirus vector, both in vitro [54,84,85,86,87] using T cell lines, PBMC and monocyte-derived macrophages, and in vivo using PBMCs [88]. To distinguish a TGS effect from a PTGS effect, we performed nuclear run-on assays, and definitively confirmed the RNA therapeutic suppressed HIV-1 via the TGS pathway [86,87]. We further confirmed there was limited PTGS contribution to HIV silencing using a 3′ LTR HIV-1 driven luciferase reporter construct [87]. We have also demonstrated the TGS mechanism involves epigenetic modifications using chromatin immunoprecipitation (ChIP) assays, which showed increased histone methylation (H3K9me2 and H3K27me3), decreased histone acetylation and recruitment of HDAC1 in the 5′ LTR promoter region [54,86,87]. Extensive investigations of PromA using several different methods have shown a distinct lack of off-target effects [85]. One explanation for not observing off-target effects for PromA, is that the sequence of the NF-κB motif targeted in HIV-1 is unique and is not homologous to any part of the human genome.

To demonstrate in vivo activity of shPromA we employed an acute model of HIV in the (NOD)/SCID/Janus kinase 3 (NOJ) knockout humanized mouse model [88]. The lentiviral-delivered shPromA was used to transduce human PBMCs, which were transplanted into NOJ mice. Following HIV-1 challenge with strain JR-FL, mice reconstituted with the anti-HIV shPromA showed significantly lower plasma viral loads and a normal CD4:CD8 T cell ratio, while the control group treated with cells transduced by the inactive mutated version of shProm A, shPromA-M2, showed the expected course of acute HIV-1 infection, with high plasma viral load and low CD4+ T cell numbers resulting in rapid onset of immunodeficiency [88]. The protective effect observed against HIV-1 likely corresponds to the “latent-like” state induced in cell line and primary cell studies, which were induced by epigenetic silencing.

Another study that has investigated an RNA therapeutic, termed S4-siRNA, specifically focussed on suppressing HIV-1 subtype C, which is prevalent in ~50% of global HIV-1 infections. The S4-siRNA was targeted to the unique subtype C NF-κB binding motif, which contains a triple repeat sequence. HIV-1 silencing was demonstrated to occur through TGS by ChIP analysis of histone methylation, which showed H3K9me2 and H3K27me3 enrichment [81]. Ex vivo suppression of HIV-1 was also demonstrated in human PBMCs transfected with S4-siRNA and then infected with different subtype C isolates [81].

The extreme breadth of sequence variation amongst HIV-1 strains is another reason for combining anti-HIV RNA targets. We have recently identified a further siRNA target, termed si143, that induces HIV-1 suppression via a similar TGS mechanism as that induced by siPromA (Figure 2b) [52]. Specifically, epigenetic profile investigations using ChIP analysis reported increased histone methylation in H3K9me3 and H3K27me3, decreased H3K9 acetylation and recruitment of Ago1 in chromatin containing the HIV-1 5′ LTR region (Figure 2c) [52]. Multiplexing of the siPromA and si143 viral targets by co-transfection demonstrated that virus silencing could not be reversed by the HDAC inhibitor vironostat (SAHA), although the highly toxic HDAC inhibitor trichostatin (TSA) did partially reverse the silencing effect [52]. Additionally, when the multiplexed shPromA/sh143 were delivered to a J-Lat 9.2 cell line viral latency model, we observed virus reactivation that was highly resistant to stimuli, including vironostat/TNF combinations (Figure 2d) [52]. This is important for a successful gene therapy strategy, whereby sustained HIV-1 suppression is essential, despite CD4+ T cell activation by homeostatic or inflammatory stimuli. Our multiplexed approach alleviates concerns regarding variation in HIV-1 sequence, as where variation occurs in the subtype C NF-κB region targeted by PromA, the 143 target sequence is conserved and would, therefore, provide protection against subtype C. We are currently testing this construct and other combinations incorporated within the lentivirus-delivered Cal-1 backbone vector in humanised mouse models. We are also investigating the potential for si/shPromA/143 to target the virus in cell types relevant to the CNS reservoir, which represent a challenging cell type to treat, due to the blood brain barrier.

## 7. RNA Therapeutics Targeting HIV by CRISPR/Cas9

Another potential future anti-HIV-1 gene therapy strategy is the use of the clustered regularly interspaced short palindromic repeats (CRISPR)-associated protein-9 nuclease (Cas9) gene editing system to excise integrated provirus DNA. There have been several studies that have reported the CRISPR-Cas9 system is efficient at excising certain regions of the provirus, which effectively disables the remaining provirus [89,90]. Liao et al. in 2014 [91] reported 5–10-fold disruption of latently integrated provirus and prolonged protection against new viral infection in the human T cell line, SupT1, stably expressing the guide RNA against LTR-R regions, gLTR-T1 and gLTR-T2, respectively. The group has also shown a three-fold reduction of virus production in human primary T cells treated with Cas9 and gLTR-T2, compared to control at 3 days post HIV-1 infection [91]. Another study by Kaminski et al. in 2016 reported the success of excising the entire HIV-1 genome (located between the 5′ LTR and 3′ LTR) in a T lymphocytic cell line, 2D10, with treated cells showing only 0.9% HIV-1 GFP reporter compared to 94.1% HIV-1 GFP positive cells in the PMA/TSA induced control [92]. Subsequently, the same lentivirus-meditated system was used to delivery Cas9/gRNA to HIV-infected CD4+ T cells from healthy individuals, and a substantial decrease in HIV-1 copy number was reported in CRISPR treated cells [92]. However, this was highly dependent on the HIV-1 strain used for infection. Experiments using the NL4-3 strain demonstrated a 210-fold reduction in HIV copy numbers to undetectable levels in all treated cells, but when the JRFL strain was used there was only a two-fold reduction in HIV-1 copy number in approximately half of the treated cells [92] This suggests that the gRNA sequence targets are not highly conserved even in these laboratory adapted B subtype strains, a common problem in highly sequence-specific approaches. The study also examined potential off-target effects in multiple genes in close proximity to the HIV-1 integration site on two separate chromosomes, and showed there were no significant effects on the levels of host RNA expression [92]. Despite these significant advances in CRISPR/Cas9 technology and relatively efficient suppression of HIV-1 replication, Wang et al. recently reported rapid and consistent generation of viral escape variants capable of evading the exquisite sequence specificity required for the CRISPR-Cas9 system [93]. This was determined by sequencing HIV-1 escape variants, which were found to be the result of errors in DNA repair by the non-homologous end-joining pathway used at the cleavage site of Cas9/guide(g)RNA [93]. Therefore, while CRISPR-Cas9 shows high potency as an anti-HIV approach, its application to gene therapy strategies will be limited until such time that the problem of virus escape can be addressed.

A modified CRISPR-Cas9 system has also been developed where the nuclease activity of Cas9 is deficient (dCas9) and fused to the VP64 transactivation domain (dCas9-VP64) [94]. This results in gene-specific transcriptional activation of promoter regions complementary to the single gRNAs. Interestingly, the hot spot identified is also the NF-κB region targeted in the aforementioned TGS RNA therapeutic approaches. The new CRISPR-mediated gene activation system may provide much needed enhanced specificity to the latency reactivation strategy and follow-up studies will show whether it is an alternate avenue for an HIV cure.

There is currently only a single clinical trial involving CRISPR/Cas9 knockout of PD-1 in autologous T cells for treatment of metastatic non-small cell lung cancer, which is due to start recruitment in late 2016 (NCT02793856). Although it does not target HIV, it will provide important information about the in vivo safety and applicability of this technology and it potential application to HIV in future clinical trials utilising the CRISPR/Cas9 system.

## 8. HIV Gene Therapy

Interest in gene therapy approaches to HIV treatment was reignited in 2009, following the remarkable success of a bone marrow transplant for acute myeloid leukemia using human progenitor stem cells (HPSC) from a CCR5-Δ32 homozygous donor. The recipient, termed the “Berlin patient”, effectively received a functional cure for HIV, as the donor HPSC were deficient for one of the HIV co-receptors, CCR5, and thus all cells that differentiated from the transplanted stem cells were protected against HIV [95]. Follow-up studies of the patient, free from ART and currently seven years post-transplantation, show no detectable plasma viral load or proviral DNA [96]. While this exceptional success has shown gene therapy has potential to treat HIV, duplicating this particular treatment has proven exceedingly difficult, with six other patients having unsuccessful transplantation of CCR5-Δ32 HPSC [96]. Europe has started a database of HLA/CCR5-Δ32 homozygous donors in the hope of increasing successful transplantations.

As described above, there are several clinical trials of gene therapy approaches targeting CCR5 in HIV-1 infected patients with non-malignancies. One completed clinical trial reported in 2014 involved gene editing of CCR5 by ZFN (SB-728mR-T) to permanently disable CCR5 expression (NCT00842634) [97]. The SB-728mR-T treatment was delivered using very large numbers of ex vivo expanded adenovirus transduced autologous CD4+ T cells into 12 patients with treated chronic HIV infection. Safety was the primary endpoint. There was one serious adverse event attributed to transfusion reaction and not the gene editing treatment, thus the use of CCR5-modified autologous CD4 T cell infusion was deemed safe [97]. The level of circulating CCR5-modified CD4 T cells at one week post-transfusion was reported to be 13.9%, however in the majority of cells there was effective disruption of only one of the two *ccr5* alleles [97]. While this provided some protection from HIV infection, it is likely that both a greater proportion of modified CD4 T cells and a higher rate of bi-allelic knock down of *ccr5* in those cells is required for any substantial effect to be observed. This is currently the study of a subsequent clinical trial (NCT02225665) to evaluate the safety and tolerability of repeat doses of SB-728mR-T following cyclophosphamide conditioning.

All current gene therapy clinical trials for HIV treatment are summarized below, separated into HIV-1 infected patients with non-malignant and malignant disease clinical trials shown in Table 1 and Table 2, respectively. Completion of these clinical trials and initiation of new clinical trials will be vital to progressing RNA therapeutics into mainstream HIV treatment.

## 9. Delivery Using Viral Vectors

**Figure 3:**
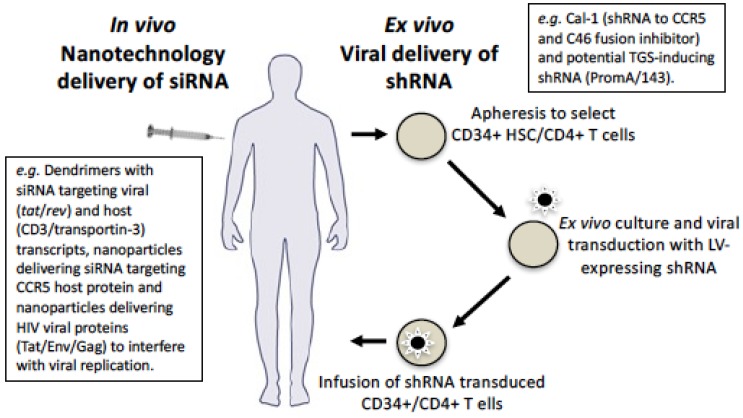
Methods of delivering si/shRNA HIV gene therapy. Current gene therapy approaches for si/shRNA targeting HIV-1 involves apheresis to obtain and select CD34+ HSC and/or CD4+ T cells that are cultured ex vivo and transduced with si/shRNAs, e.g. the Cal-1 LV construct containing CCR5 shRNA and C46 fusion peptide inhibitor and our envisaged use of TGS-shRNAs PromA/143. Transduced cells can then be infused back into the patient. Alternately, siRNA can be packaged in a nanoparticle and delivered in vivo or ex vivo. This has been done in vivo via intravenous administration for (i) cationic PAMAM dendrimers encapsulating siRNA targeting viral (*tat*/*rev*) and host (CD3 and transportin-3) transcripts; (ii) nanoparticles to deliver siRNA targeting CCR5 host protein; and (iii) nanoparticles to delivery HIV viral proteins (Tat/Env/Gag) to interfere with viral replication. Ex vivo delivery of Cal-1 is being trialled and we envisage the same approach with our TGS-inducing shPromA/143. Both in vivo and ex vivo delivery pathways will provide protection against HIV-1 by targeting various virus and host factors.

Delivery is one of the most challenging aspects of developing an effective gene therapy strategy, irrespective of the gene or disease being targeted. Currently the dominant delivery method for therapeutics in clinical trials is large-scale apheresis followed by autologous re-infusion of ex vivo modified hematopoietic stem cells (HSC) and/or CD4+ T cells transduced with a viral vector, usually a lentivirus, adenovirus, or adeno-associated virus (AAV) [100] (Figure 3). However, alternates to this are being explored, particularly the challenging goal of in vivo delivery, which will also be discussed below.

Ex vivo delivery by the aforementioned viral vectors involves RNA polymerase III promoter driving expression of shRNA transcripts that form a single-strand hairpin loop, which is then exported out of the nucleus and processed in the cytoplasm to form mature siRNA, which can then enter the PTGS and/or TGS pathways (see Figure 1). Lentivirus vectors integrate and, relative to γ-retroviral vectors, have a lower propensity to cause insertional oncogenesis [101]. Their integrative property can aid in increasing the longevity of therapeutic delivery. It is interesting that many gene therapies use a lentivirus-based vector, comprised of a partial HIV-1 genome. This presents some potential disadvantages in its use to target HIV, as transduction levels may be decreased due to shRNA targets also being present in regions encoded on helper plasmids, such as *gag, pol*, and *rev* [102]. Further, to improve safety of lentiviral vectors, the native HIV-1 envelope is deleted and a separate plasmid encoding the vesicular stomatitis virus glycoprotein (VSV-G) is incorporated. While VSV-G has a broad cell tropism, including CD34+ HSC, it does not have the ability to fuse and enter the host cell plasma membrane of resting CD4+ T cells, which represent a large proportion of the cells harbouring the HIV-1 latent reservoir. Various approaches are being explored to alleviate this disadvantage, including incorporating an envelope from other virus types to improve viral entry.

Another disadvantage of using current lentiviral vectors in the context of HIV treatment is that ART can interfere with virus transduction levels in target cells, due to the drugs specifically targeting HIV proteins required for lentivirus transduction. This was recently reported in the pigtail macaque SHIV model [103], which is the same model described above for the recent in vivo studies of Cal-1, without ART suppression. In this model, ART (raltegravir, emtricitabine, and tenofovir) is used to stably suppress SHIV-infection, at which stage an autologous CD34+ HSC transplantation is performed using lentiviral transduced cells expressing the anti-HIV C46 fusion inhibitor [103]. The study reported that following transplantation, SHIV-infected and ART-treated animals displayed extremely low gene marking levels, likely contributed to by the three ART drugs [103]. Indeed, mass spectrometry/HPLC analysis confirmed the presence of the three ART drugs in CD34+ and PBMCs directly prior to lentivirus transduction [103], providing strong evidence that the residual intracellular ART-mediated inhibition of lentiviral reverse transcription and/or integration was responsible for the highly inefficient lentiviral transduction efficiency. This study suggests that an extended ART interruption time or the use of protease inhibitors is required for efficient lentivirus transduction of target cells to occur [101] and represents another hurdle for HIV treatment delivery. Use of alternate vector delivery systems, which are not affected by ART, may be one possible way to avoid the complications associated with patients undergoing ART interruption.

## 10. Delivery Using Nanotechnology

While viral vectors have proven to be effective si/shRNA carriers [104], their immunogenicity, toxicity, poor scalability, potential to induce insertion mutagenesis, and limited capacity to transduce resting T cells and myeloid cells has motivated research into safer, biocompatible, non-viral alternatives. Various non-viral strategies for si/shRNA delivery are currently under investigation [105,106] and include: (i) chemical modification of siRNA [107,108,109,110,111]; (ii) conjugation of siRNA to small ligands [112,113,114], e.g., cholesterol and peptides; and (iii) the incorporation of siRNA within nanoparticles based on lipids [115,116,117], biocompatible polymers/dendrimers [118,119,120,121], polypeptides [122,123], and inorganic materials [64,124,125,126,127,128,129,130,131]. Chemical modification and conjugation of ligands can increase the enzymatic stability of siRNA and prevent RNase cleavage. However, rapid renal filtration and elimination are not avoided. Packaging siRNA within nanoparticles not only protects it from nucleases, but also shields its overall negative charge, which can prolong the in vivo circulation life in blood and facilitate its passage across the cell membrane to the cytoplasm of target cells [105,106].

The success of nanoparticle-based RNAi delivery is highly dependent on several factors that affect RNAi efficiency, including the route of administration, nonspecific immune activation, circulation time, tissue extravasation, targeting, cell internalization, endosomal escape, and off-target effects. Despite these difficulties, nanoparticles are ideally suited to deliver siRNA in complex biological milieu due to their chemical and structural versatility and specificity. Smart and stimuli-responsive nanoparticles can be engineered to improve delivery efficiency and efficacy. In addition, some nanomaterials have therapeutic effects by themselves. In fact, various nanomaterials have been found to inhibit viral replication in vitro and it is suggested that these effects are based on structural interference with viral assembly [132].

The first published nanoparticle-based siRNA delivery system trialled in humans was based on targeted cyclodextrin and was delivered via systemic administration for cancer therapy [133]. Although the study showed modest results, it highlighted the clinical significance of non-viral siRNA delivery and the challenges that must be overcome to improve their efficacy. To date, there are no approved siRNA-based therapies in the clinic, but a number of nanoparticle systems are under clinical trials for various diseases and infections, including cancer, macular degeneration, respiratory syncytial virus (NCT01065935), influenza (NCT01747148), hepatitis B and C, Ebola, and HIV-1 [100,134].

There is remarkable potential for the application of siRNA-loaded nanoparticles for the treatment and cure of HIV, but this is not without challenges [132,135,136]. As stated above transfection to primary immune cells, especially when they are in their resting state, is difficult. Nanoparticles designed to improve delivery to T cells have exploited the use of antibodies against surface markers, including anti-CD7, conjugated to solid chitosan particles [137]. Peer and co-workers decorated lipid nanoparticles with anti-CD4 antibody, leading to efficient binding and uptake by CD4+ T cells, and robust siRNA-induced silencing of CD45 [138]. Lipid nanoparticles containing mixtures of lipids, including fusogenic and ionizable amino lipids, were prepared to enhance both the encapsulation of siRNAs and endosomal escape once delivered to the target cells. Importantly, CD45 silencing was restricted to the CD4+ T cells and was not observed in other lymphocyte subsets. As described above, other PTGS RNAi targets for gene silencing in HIV have been investigated, including the CCR5 host protein to confer HIV-1 resistance as delivered by nanoparticles [139], and HIV viral proteins including Tat, Env, and Gag to interfere with viral replication [140]—both of which were delivered by nanoparticles. However, to date, studies investigating delivery of nanoparticle-mediated TGS-inducing RNA therapeutics have not been reported. This represents an important and currently unexplored alternate avenue for HIV treatment.

The application of delivery carriers, primarily based on lipids and polymers, in HIV therapy also paves the way for multifunctional vectors carrying a combination of agents that can work synergistically to improve the treatment of HIV. One example of this developed by Rossi and co-workers is cationic poly(amidoamine) (PAMAM) dendrimers, which encapsulate a cocktail of siRNA targeted to both viral (*tat/rev*) and host (CD4 and transportin-3) transcripts [141]. Delivery via intravenous injection in HIV-1 infected humanized mice led to a reduction in viral RNA load. Opportunities for the delivery of RNA therapeutics targeting PTGS and TGS, as well as other druggable targets, are potentially adaptable to a nanoparticle carrier platform. 

Important aspects to consider in the application of nanotechnology-based delivery of RNA therapeutics for HIV treatment include route of administration and economic aspects, particularly as the global HIV burden and most vulnerable populations reside in the resource-poor developing world. In this context, in addition to injectable administration as depicted in Figure 3, RNA-nanotherapeutics delivered via oral, transdermal, nasal, and pulmonary routes should also be investigated, considering the ease of administration and lower cost compared to ex vivo delivery. Finally, when further optimised, RNA-nanotherapeutics may have the potential to reduce dosing frequency and eradicate or permanently silence viral reservoirs. These advantages could effectively offset the costs of fabricating nanomaterials.

## 11. Future Perspectives

Gene therapy approaches for HIV-1 treatment have advanced dramatically since the first reported success nearly a decade ago. Similarly, promising RNA therapeutics have and will continue to develop utilizing both PTGS and TGS pathways, as well as novel CRISPR/Cas9 and dCas9(VP64) systems. The current hurdle for gene therapy strategies lies in the delivery stage, where significant leaps in technology must be achieved for gene therapy constructs to become a practical alternative approach to controlling HIV-1 infection.

## Figures and Tables

**Table 1:** Gene therapy clinical trials for HIV-1 infected patients with non-malignant disease.

Gene Therapy Trials	Intervention/s	Target/s	Identifier	Sponsor/Collaborator	Stage/Status
Dual Anti-HIV Gene Transfer Construct, LVsh5/C46 (Cal-1)	CCR5 shRNA C46 peptide Busulfan	Host co-receptor Viral Env	NCT01734850	Calimmune, Inc.	Phase I/II Recruiting
Long Term Follow up of Delayed Adverse Events in Cal-1 Recipients	Blood tests for general health, complete blood count and Cal-1 specific analyses		NCT02390297	Calimmune, Inc.	Recruiting by invitation
Redirected MazF-CD4 Autologous T-Cells	CCR5 MazF	Host co-receptor	NCT01787994	University of Pennsylvania	Phase I Ongoing
T-Cells Modified at CCR5 Gene by ZFN SB-728mR	CCR5 ZFN	CCR5 DNA	NCT02388594	University of Pennsylvania/NIAID	Phase I Recruiting
SB-728mR-T After Cyclophosphamide Conditioning	CCR5 ZFN	CCR5 DNA	NCT02225665	Sangamo Biosciences	Phase I/II Ongoing
Autologous T-Cells Modified at CCR5 Gene by ZFN SB-728	CCR5 ZFN	CCR5 DNA	NCT00842634	University of Pennsylvania/ Sangamo Biosciences	Phase I Completed
Redirected High Affinity Gag-Specific Autologous T Cells	WT-gag-TCR or α/6-gag-TCR	CD8 TCR	NCT00991224	University of Pennsylvania/ Adaptimmune	Phase I Completed
*This study was closed before any patient received T cells transduced with a high affinity A2-SL9-specific TCR [98]*					
Autologous CD34+ HSCs Transduced With Anti-HIV-1 Ribozyme (OZ1)	Tat-vpr ribozyme	Tat-vpr mRNA	NCT00074997	Janssen-Cilag Pty Ltd.	Phase II Completed
Long Term Follow-Up Study of OZ1 Gene Therapy	Blood tests for quantitative marking of the gene transfer product in PBMCs over time		NCT01177059	Janssen-Cilag Pty Ltd.	Phase II Recruiting by invitation
Tolerability and Therapeutic Effects of Repeated Doses of Autologous T Cells With VRX496	VRX496 antisense RNA	Env mRNA	NCT00295477	University of Pennsylvania/NIAID	Phase I/II Ongoing
Safety and Efficacy of T-Cell Genetic Immunotherapy	VRX496 antisense RNA	Env mRNA	NCT00131560	VIRxSYS Corporation	Phase II Ongoing

Abbreviations: ZFN, zinc finger nuclease; Env, envelope; WT, wild-type; TCR, T cell receptor; and NIAID, National Institute of Allergy and Infectious Diseases [99].

**Table 2:** Gene therapy clinical trials for HIV-1 infected patients with malignant disease.

Gene Therapy Trials	Intervention/s	Target/s	Identifier	Sponsor/Collaborator	Stage/Status
L-TR/Tat-neo in Patients With Non-Hodgkin’s Lymphoma	Tat ribozyme	Tat-rev mRNA	NCT00002221	Ribozyome	Phase II Completed
M87o autologous HSCs for Patients with Malignant Diseases	C46 peptide	Viral Env	NCT00858793	University Medical Center Hamburg-Eppendorf	Phase I/II Suspended
*A leukaemia case was reported in patient treated with a similar vector. For safety risk recruitment was stopped.*					
C46/CCR5/P140K modified autologous HSCs in patients with lymphoma	C46 peptide, CCR5 ribozyme, MGMT^P140K^ mutant	Viral Env, CCR5 mRNA, Alkylating agent resistance	NCT02343666	Fred Hutchinson Cancer Research Center/NCI/NHLBI	Phase I, Not yet recruiting
Autologous Transplantation of HSCs With LVsh5/C46 (Cal-1) for Treatment of HIV-Related Lymphoma	CCR5 shRNA, C46 peptide	Host co-receptor, Viral Env	NCT02378922	Fred Hutchinson Cancer Research Center/NCI	Phase I Recruiting
rHIV7-shI-TAR-CCR5RZ-transduced HSC in patients with AIDS-related Non-Hodgkin Lymphoma	tat/rev shRNA, TAR decoy, CCR5 ribozyme, Busulfan	Viral mRNA, Viral tat proteinm, CCR5 mRNA, Transplant conditioning	NCT02337985	City of Hope Medical Center/NCI	Pilot Recruiting
rHIV7-shI-TAR-CCR5RZ-transduced HSC in patients with AIDS-related non-Hodgkin’s lymphoma	tat/rev shRNA, TAR decoy, CCR5 ribozyme, Busulfan	Viral mRNA, Viral tat protein, CCR5 mRNA	NCT01961063	City of Hope Medical Center	Pilot Recruiting
rHIV7-shI-TAR-CCR5RZ-transduced HSC in patients undergoing stem cell transplant for AIDS-related lymphoma	tat/rev shRNA, TAR decoy, CCR5 ribozyme, Busulfan	Viral mRNA, Viral tat protein, CCR5 mRNA, Transplant conditioning	NCT00569985	City of Hope Medical Center/NCI	Pilot, Ongoing
shRNA/TRIM5alpha/TAR Decoy-transduced Autologous HSC in Patients With HIV-Related Lymphoma	CCR5 shRNA, RNF88, TAR decoy	Host co-receptor, Gag p24, Viral tat protein	NCT02797470	AIDS Malignancy Consortium/NCI	Phase I/II

Abbreviations: HSC, hematopoetic stem cell; MGMT, O^6^-methylguanine DNA methyltransferase; NCI, National Cancer Institute; NHLBI, National Heart, Lung and Blood Institute; NIAID, National Institute of Allergy and Infectious Diseases; RNF88, RING finger protein 88; shRNA, short hairpin RNA; and ZFN, zinc finger nuclease [99].
